# The Chicago Thoracic Oncology Database Consortium: A Multisite Database Initiative

**DOI:** 10.7759/cureus.533

**Published:** 2016-03-16

**Authors:** Brian Won, George B Carey, Yi-Hung Carol Tan, Ujala Bokhary, Michelle Itkonen, Kyle Szeto, James Wallace, Nicholas Campbell, Thomas Hensing, Ravi Salgia

**Affiliations:** 1 Section of Hematology/Oncology, The University of Chicago Medicine; 2 The Perelman School of Medicine, University of Pennsylvania; 3 Section of Hematology/Oncology, NorthShore University HealthSystem; 4 Feinberg School of Medicine, Northwestern University; 5 MrktServ, LLC; 6 Oncology-Hematology, Ingalls Health System; 7 Section of Hematology/Oncology, NorthShore University Health System; 8 Pathology and Dermatology, The University of Chicago Medicine

**Keywords:** database, bioinformatics, lung cancer

## Abstract

Objective: An increasing amount of clinical data is available to biomedical researchers, but specifically designed database and informatics infrastructures are needed to handle this data effectively. Multiple research groups should be able to pool and share this data in an efficient manner. The Chicago Thoracic Oncology Database Consortium (CTODC) was created to standardize data collection and facilitate the pooling and sharing of data at institutions throughout Chicago and across the world. We assessed the CTODC by conducting a proof of principle investigation on lung cancer patients who took erlotinib. This study does not look into epidermal growth factor receptor (EGFR) mutations and tyrosine kinase inhibitors, but rather it discusses the development and utilization of the database involved.

Methods:  We have implemented the Thoracic Oncology Program Database Project (TOPDP) Microsoft Access, the Thoracic Oncology Research Program (TORP) Velos, and the TORP REDCap databases for translational research efforts. Standard operating procedures (SOPs) were created to document the construction and proper utilization of these databases. These SOPs have been made available freely to other institutions that have implemented their own databases patterned on these SOPs.

Results: A cohort of 373 lung cancer patients who took erlotinib was identified. The EGFR mutation statuses of patients were analyzed. Out of the 70 patients that were tested, 55 had mutations while 15 did not. In terms of overall survival and duration of treatment, the cohort demonstrated that EGFR-mutated patients had a longer duration of erlotinib treatment and longer overall survival compared to their EGFR wild-type counterparts who received erlotinib.

Discussion: The investigation successfully yielded data from all institutions of the CTODC. While the investigation identified challenges, such as the difficulty of data transfer and potential duplication of patient data, these issues can be resolved with greater cross-communication between institutions of the consortium.

Conclusion: The investigation described herein demonstrates the successful data collection from multiple institutions in the context of a collaborative effort. The data presented here can be utilized as the basis for further collaborative efforts and/or development of larger and more streamlined databases within the consortium.

## Introduction

As healthcare centers continue to move to the use of electronic medical records, a plethora of data will become more readily available to physicians, researchers, and others in the medical field. This development stands to be a huge benefit to researchers, as it will enable them to undertake increasingly sophisticated investigations more easily. However, in order to take advantage of improved data availability, we must first create effective systems to extract, store, utilize, and protect this information with thoughtfully designed disease-specific databases and informatics infrastructures. Beyond just the creation of these databases, however, an issue of paramount importance is that multiple research groups must be able to coordinate their collection of this data so that they can collect and share data in an efficient and effective manner: data elements must be standardized, informatics platforms must be able to communicate, and institutions must develop data sharing agreements that facilitate efficient and ethical data flow between collaborators.

To address this issue in thoracic oncology, colleagues at The University of Chicago Thoracic Oncology Research Program (TORP) have organized the Chicago Thoracic Oncology Database Consortium (CTODC). The CTODC is a collection of research groups within Chicago that have agreed to follow common database practices in order to enable better data sharing and improved translational research.

In this paper, we assess the CTODC’s database infrastructure and data sharing model. In order to do so, we perform a proof of principle investigation into patients with lung cancer receiving erlotinib at three institutions belonging to the CTODC: The University of Chicago Medicine, Ingalls Health System, and NorthShore University Health System.

### Background: EGFR and erlotinib in lung cancer

Lung cancer is the leading cause of cancer death among both men and women in the United States, with about 221,000 new cases and an estimated 158,000 deaths in 2015 [1]. Lung cancer can be subdivided into histological subtypes: small-cell lung cancer (SCLC), which comprises 15% of lung cancer, and non-small cell lung cancer (NSCLC), which comprises 85% of lung cancer [2]. NSCLC can be further categorized as adenocarcinoma, squamous cell carcinoma, and large-cell carcinoma [3]. Patients often present with metastatic disease, and if left untreated, have a median survival time of four to five months after diagnosis and a five-year survival rate of less than 15% [4]. In order to curb poor prognosis, research has progressed to assess genetic abnormalities of tumors in individual patients, giving rise to personalized molecular marker therapeutics [5]. In this light, various molecular abnormalities were identified as key players in the malignancy of lung cancers, one of which is mutations in the epidermal growth factor receptor (EGFR) [6]. EGFR belongs to a family of receptor tyrosine kinases (RTKs), which serve as mediators of cell growth and reproduction through extracellular growth factors [7]. Overexpression and mutations of this RTK, especially in its tyrosine kinase domain, are closely linked to poor prognosis [8-10]. In the context of lung cancer, EGFR mutations are more common in adenocarcinomas, East Asian populations, women, and never smokers [11]. Although over 100 mutations have been identified in EGFR mutated patients, only two account for 85% of EGFR mutations: exon 19 deletions and the L858R point mutation in exon 21 [12].

One of the most commonly used targeted therapeutic agents for EGFR mutated lung cancers is erlotinib (Tarceva). Erlotinib is a reversible inhibitor of the EGFR kinase and competitively inhibits ATP-binding at the active site of the EGFR kinase domain [13-14]. Erlotinib and other similar therapeutic agents, such as gefitinib, have proven to be effective with a ~75% response rate for EGFR mutant NSCLCs [15]. Unfortunately, most patients develop therapeutic resistance to either drug six to 12 months after their initial treatment [2]. This resistance has been attributed to acquired and innate mutations in EGFR and other molecular markers; one such mutation is T790M mutation in exon 20 of EGFR [16]. This mutation is found in 60% of patients with an acquired resistance, but it can also be found prior to treatment with EGFR inhibitors [17-19]. The mutation confers resistance by lowering the growth kinetics in tumor cells, which weakens the binding between EGFR inhibitors and their target [20]. Currently, various strategies are being developed to overcome this resistance, such as irreversible inhibitors, which covalently bind to EGFR and combination therapies, which pair conventional EGFR inhibitors with drugs that target alternative resistance pathways [12].

We undertook a study to determine the feasibility of pooling data as well as responsiveness to erlotinib in patients with non-small-cell lung cancer. We started to collect this data even before the genetic analysis of EGFR became the standard of care. This study is not to emphasize the EGFR mutation and role of tyrosine kinase inhibition, but rather to detail the database developed as well as the utilization of the database.

## Materials and methods

### Patient enrollment criteria

Patients at all three sites of the CTODC were enrolled on IRB approved protocols. The University of Chicago created and implemented the 13473A, which Ingalls implemented as well. NorthShore developed and used the EH98-136 as its IRB approved protocol.

The University of Chicago

Inclusion Criteria: Patients were included if they were under the care of a University of Chicago Medicine oncologist for a pathologically diagnosed lung cancer. Patients must have received erlotinib in the course of their treatment. No healthy controls were included. All patients were adults.

Subject Enrollment: All patients at The University of Chicago were enrolled on at least one of three protocols approved by The University of Chicago Institutional Review Board (IRB): IRB 9571, 13473A, and/or 10-654N. Under 9571, patients sign a consent for prospective banking of biospecimens (blood, tissue, and/or sputum), as well as data collection via patient interview and chart abstraction. Patients enrolled on 13473A have signed a consent to allow data banking and banking of samples previously acquired in the course of clinical care. IRB 10-654N is a consent-waived protocol that authorizes the use of previously collected biospecimens of deceased patients, as well as associated clinical and demographic data. These protocols also allow for collaboration with outside researchers through the use of de-identified data sets.

Ingalls Health System

Patients were consented under an IRB-approved protocol from the University of Chicago, IRB 13473A. Again, the 13473A allows data banking and banking of samples previously acquired throughout the course of patients’ clinical care.

NorthShore University Health System

Patients were consented to the study EH98-136: “The Establishment and Maintenance of a Comprehensive Thoracic Tumor Data Registry and Biorepository.” EH98-136 is a comprehensive thoracic cancer data registry that collects clinical and pathologic data prospectively for all consented subjects who have clinically or pathologically confirmed disease to provide the opportunity for the further in-depth study of these malignancies. The registry collects information about the subject’s demographics, co-morbidities, diagnostic test results, tissue histology, molecular markers and classification, surgical/chemotherapy/radiation therapy treatments, and pertinent side effects with detailed specimen tracking. Follow-up information to determine disease status and survival is through the review of the medical records and possible phone or mail contact with the subject and/or family. The Thoracic Biorepository is a repository of tumor specimens to include both normal and cancerous thoracic tissue (including lung, mediastinal, esophageal, mesothelial, and thymic tissues) and associated lymph nodes removed at the time of thoracic surgery and/or biopsy as well as bodily fluid samples, such as blood, saliva, sputum, and/or pleural fluid.

### Informatics infrastructures

**Figure 1:**
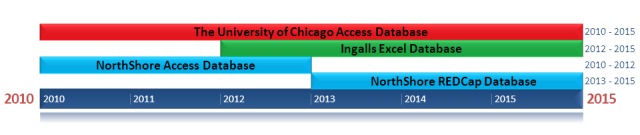
Timeline of database implementation at each institution within the CTODC The timeline describes how long databases were implemented for at each institution. It does not describe the time range of patient data collection at each institution.

All three institutions of the consortium implement their own primary databases. The University of Chicago uses the Microsoft Access Database, which has been implemented since 2010. Ingalls Health System uses the Microsoft Excel Database, which has been implemented since 2012. NorthShore University Health System has utilized two different databases since 2010: The Microsoft Access Database (2010 - 2013) and the Research Electronic Data Capture Platform (REDCap) Database (2013 - Current). These timelines are displayed in Figure 1.

Database Platforms

**Figure 2:**
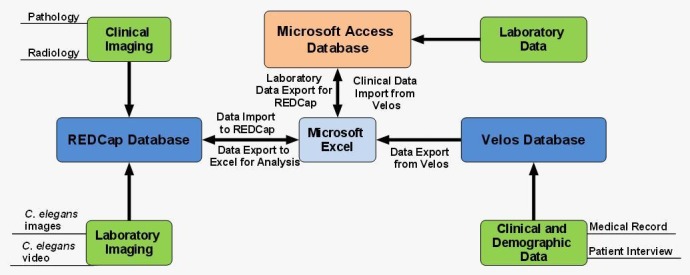
Mind map illustrating the relationship among REDCap, eVelos, and Microsoft Access databases This schematic illustrates the relationship between different databases described within the University of Chicago Thoracic Oncology Database SOP.

The University of Chicago: The University of Chicago Thoracic Oncology Research Program (TORP) created the Thoracic Oncology Program Database Project (TOPDP) Access database to capture translational research data. This database has been of great utility for the Salgia lab and has facilitated significant translational research [21]. Nevertheless, we sought to expand the capabilities of our database design by implementing the TORP Velos database, an online Oracle-based system that enables remote access to data and which is also used by The University of Chicago for clinical trial patient data collection and reporting. In addition, we have since implemented the TORP REDCap database in order to capture radiology and pathology images [22]. The TOPDP Access database serves as the central data repository; data are transported between the databases with the use of Excel and original automated Visual Basic for Applications (VBA) scripts, which are run in the TOPDP Access database. Detailed information on each database can be found on the iBridge network [23]. Figure 2 presents a chart detailing the database relationships at The University of Chicago.

**Figure 3:**
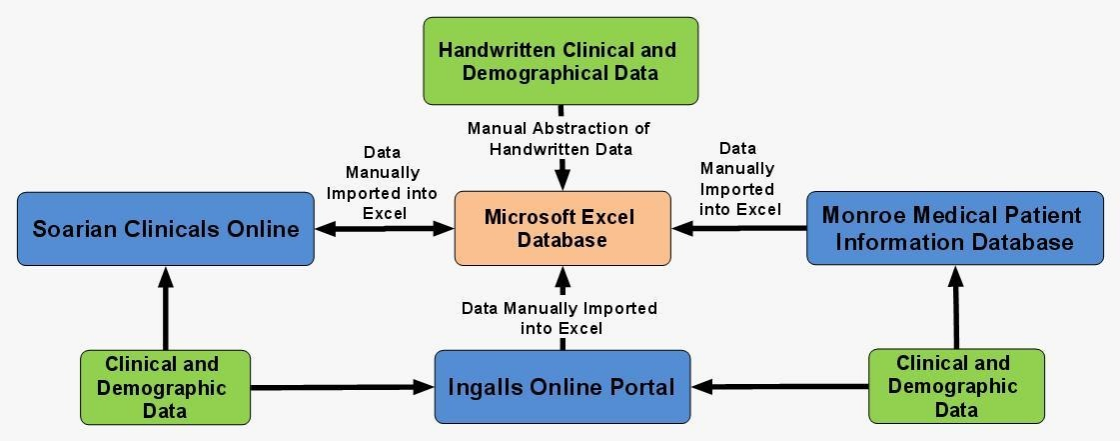
Mind map illustrating database relationships at Ingalls Health System

Ingalls Health System: The Ingalls Health System database for patients with NSCLC was created using Microsoft Excel based on the SOP for the TOPDP Access database [24]. The Ingalls database infrastructure is shown in Figure 3.

**Figure 4:**
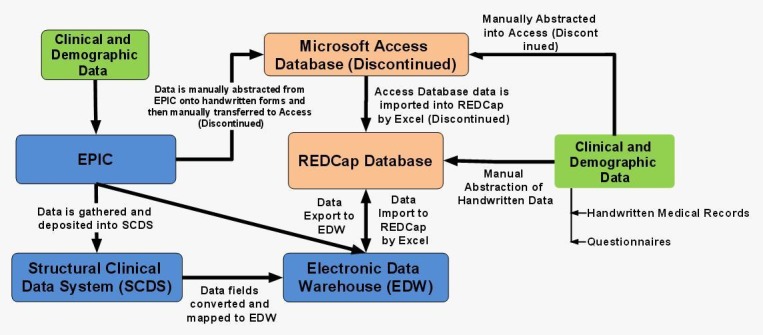
Mind map illustrating database relationships at NorthShore University Health System

NorthShore University Health System: Before February of 2013, NorthShore entered data in The Thoracic Tumor Data Registry, which utilized the 2010 Microsoft Access database. The Thoracic Tumor Data Registry was created based on the licensed TOPDP Access database SOP [24]. NorthShore eventually developed and implemented the Thoracic Tumor Data Registry in REDCap based on the TORP REDCap SOP as well [25]. In this new format, data is housed in the REDCap web-based research database suite that is HIPAA-compliant and password-protected. This program has the ability to de-identify data for export and statistical analysis to research collaborators. Data from the Microsoft ACCESS database is conveniently exported to REDCap without any loss of information. NorthShore currently uses the Electronic Data Warehouse (EDW) to link clinical patient chart notes to the research file. In addition, NorthShore uses a Structural Clinical Data System (SCDS) for Thoracic Oncology in the EMR (electronic medical record), EPIC (Epic Systems Corp.). This system allows for thoracic oncology medical notes - which are written in real-time as part of standard documentation - to gather specific research data elements as non-free-text entries and then deposit this information directly into the EPIC EMR, REDCap, EDW and the Microsoft ACCESS databases. This provides an efficient means of data collection that can then be easily shared with collaborators. Relationships between the database infrastructures at NorthShore are presented in Figure 4.

Data Elements

The core data elements collected by the CTODC are presented in The University of Chicago SOPs and have been formulated based on the NCI Common Data Elements Committee. However, additional variables of interest have been identified by a multidisciplinary panel of thoracic oncology researchers and are therefore also collected. While all members of the CTODC have committed to collecting core data elements, each institution is free to expand upon these variables as desired.

Data Collection

**Figure 5:**
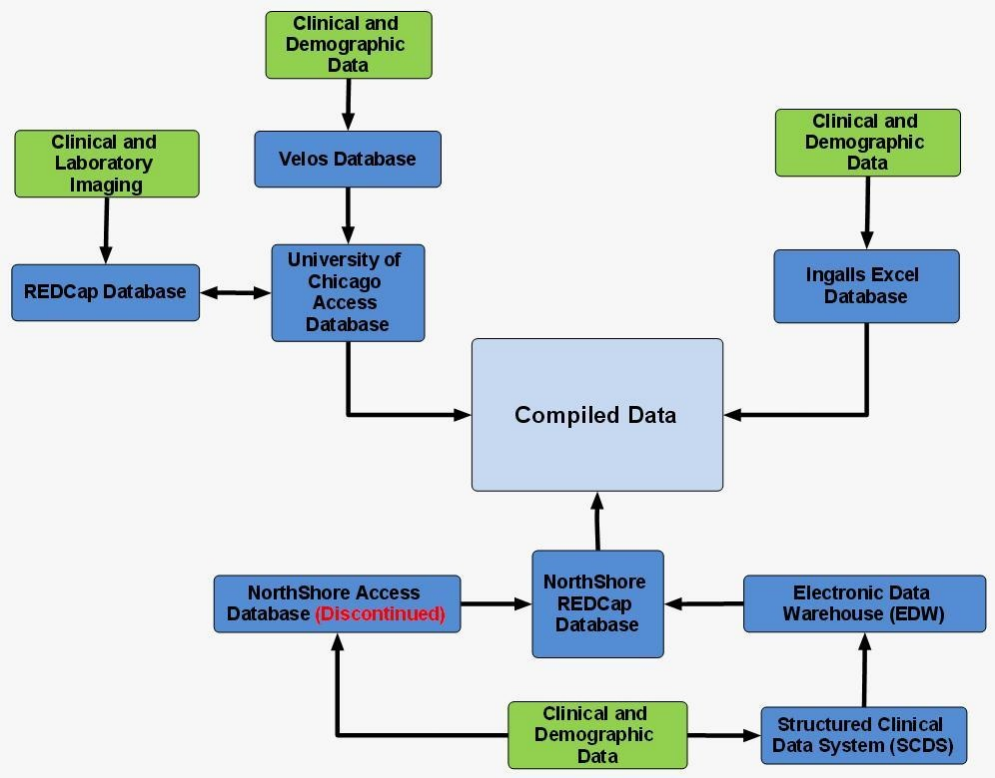
Mind map illustrating the relationships between the consortium databases utilized for this project

Data is collected separately at each institution. For the purpose of this project, data was first collected normally at each institution and then compiled. Figure 5 presents a flow chart that visually details the data flow at each institution and how data from each institution ends up compiled.

The University of Chicago: Patient information was collected and entered by research assistants (RAs) with The University of Chicago Thoracic Oncology Program. RAs consented patients during clinic visits and interviewed patients regarding their demographic and relevant clinical data, such as diagnostic and treatment history. Information obtained via interview was supplemented by chart review of The University of Chicago EMR system. Chart abstraction is also used to verify data obtained during patient interviews. Patient follow-up and outcomes data are collected by periodic chart review and consultation of the Social Security Death Index.

Ingalls Health System: Patients are consented by a research nurse who contacts patients and informs them of the study. Once patients give consent, the research nurse then abstracts the data onto case report forms (CRFs), and the data is then manually entered into the Microsoft Excel spreadsheet database by the data manager.

The Ingalls medical record system used to collect and centralize patient information in 2010 included handwritten patient charts, the Ingalls Online Portal, and Soarian Clinicals Online. The 2012 procedure involved updating patient information since 2010 with the purpose of including additional fields in adherence with the updated 2012 TORP Comprehensive Standard Operating Procedure. Next, new patients who had been diagnosed with NSCLC since 2010 were added to the 2012 Ingalls Excel NSCLC database. This involved collection of patient information from physical patient charts, Ingalls Portal, Soarian Clinicals, as well as the use of a modern database, the Monroe Medical Patient Information Database, which has been implemented since 2010.

NorthShore University Health System: Previously with ACCESS database, data was manually abstracted. The research coordinator abstracted data onto CRFs. The data was then manually entered into the ACCESS database by the research coordinator. Summer research interns were also utilized to help with data abstraction and data entry.

As of March 31^st^, 2014, all data are now transferred to the REDCap database. Since the SCDS project went live around December 2013, data abstraction has become less time-consuming and more convenient. Therefore, the use of hard copies was eliminated, and data was then abstracted manually from the patient electronic medical records to the REDCap database. 

Data Sharing

In this investigation, data at NorthShore were collected and then de-identified by NorthShore researchers before being sent off to The University of Chicago TORP. However, the data at Ingalls Health System were collected but not de-identified before being transferred to the University of Chicago. Data were then compiled and analyzed at the University of Chicago using Microsoft Access and Microsoft Excel. Specific details regarding data sharing agreements and practices for each institution are presented below.

NorthShore University Health System and The University of Chicago: The process is detailed, extensive, and goes through various levels of security. The NorthShore Data Governance Committee has been created to protect NorthShore’s data by reviewing and approving the extraction of data from NorthShore’s production systems. Two new policies have been created: AD-8060 - “Removal or Extraction of Protected Health Information (PHI) or Sensitive Information,” and AD-8061 - “Encryption of Data.” These policies relate to electronic data containing PHI or sensitive information that is to be released to third parties and/or is to be extracted from a production system (including, but not limited to Epic, Enterprise Data Warehouse, Lawson, PeopleSoft, etc.) to a non-production system (including, but not limited to reports, queries, databases, Excel, Access, case report forms, etc.).

The form is electronically submitted to the Data Governance Committee. The research coordinator needs to secure approval from the NorthShore Data Governance Committee prior to removing or extracting PHI or proprietary data. The committee ensures that all released data is encrypted according to the Administrative Policy AD-8061 while in transit from NorthShore via any means—email, flash drive, laptop, file transfer server, etc.

The final step is to set up a user account on the File Transfer Protocol (FTP) server. The FTP server accounts are set up for the study coordinator and the University of Chicago collaborator, allowing for sharing of data on the server that can be seen and then downloaded accordingly.

Ingalls Health System and The University of Chicago: Since patients at Ingalls are consented onto the 13473A, a tissue banking study that originates from the University of Chicago, it is unnecessary to have patients be de-identified prior to the transfer of data. As a result, none of the patients’ data are modified in any way. The data are transferred to a Microsoft Excel spreadsheet from the data manager at Ingalls to the data manager at the University of Chicago.

EGFR Testing Sources and Methods

All three institutions of the CTODC collect EGFR mutation statuses of their lung cancer patients if testing was performed. However, testing methods and sources vary at different institutions. At the University of Chicago, all of the EGFR tests were performed by Foundation Medicine, Response Genetics, and Caris, which are all outside molecular testing companies. At the time the patient cohort was accrued for this study, Foundation Medicine and Caris used next-generation sequencing (NGS) for testing EGFR mutations while Response Genetics used a polymerase chain reaction (PCR)-based assay. None of the EGFR testings for the University of Chicago cohort was done on site at the University of Chicago. For NorthShore, EGFR testing was performed on site by using a PCR-based assay. This assay was designed to detect mutations in exon 19 and 21 of the EGFR gene by amplifying regions of the EGFR gene encompassing codons in exon 19 and exon 21. Additional assays were then performed to make the final determination of the EGFR mutation status. For the Ingalls patient cohort, EGFR testing was done entirely through Response Genetics.

Database Elements and Development

The database infrastructures implemented by the institutions of the CTODC are based off the University of Chicago TORP database SOP in order to standardize data collection.

The primary database platform, the Microsoft Access Database, was chosen to fill its role due to its relational nature, ease of deployment, use, and customization. Its low cost and ubiquity are also ideal attributes and enable collaboration between different institutions worldwide. Moreover, Microsoft Access offers researchers a stable platform, which assures researchers that their copy will not be taken away due to lack of funding or by an outside institution or board.

However, Microsoft Access has some issues. Some of these problems are remediable, such as the lack of security. Although Access lacks inherent security features that are compliant with the Health Insurance Portability and Accountability Act (HIPAA) Security and Privacy Rules, a combination of optional Access security features and Visual Basic for Applications (VBA) scripts have remedied this issue.

There are some limitations that are not remediable, such as Microsoft Access’ 2 gigabyte (GB) file size limit. This limitation led to the search for other platforms which could resolve this issue. One solution was found by implementing Velos, an Oracle-based program already in use by the University of Chicago Medical Center for clinical trials research. This program was integrated into the TORP database SOP by developing thoracic oncology-specific data capture forms, which record more detailed information than the Access database and allow for multiple data points to be captured for a particular variable.

While Velos met some of the needs for translational research, it did not meet all of them. For example, Velos could not capture and store large files (e.g., medical images). For this reason, the TORP database integrated the REDCap platform. REDCap includes the inherent security measures required by HIPAA; provides a user interface, which facilitates easy development and customization of data capture forms; and affords users 1 terabyte (TB) of storage space, making it ideal for storage of large files. However, REDCap is not relational.

All three databases within the University of Chicago TORP SOP possess their own strengths and weaknesses. However, by implementing them in conjunction, each database platform’s weaknesses are supplemented by the strengths of the other platforms.

## Results

### Patient characteristics

After querying data from all three institutions of the CTODC, 373 patients who took erlotinib anytime between 2005 and 2013 were identified. Out of these total patients, 296 were from the University of Chicago, 48 were from the NorthShore, and 29 were from Ingalls. In the total patient pool, 188 were Caucasian, 124 were African-American, 23 were Asian, and one was an American Indian/Alaskan Native. One was Native Hawaiian/Pacific Islander and 36 were unknown. In regards to ethnicity, 327 were reported not to be Hispanic/Latino, while only three reported to be Hispanic/Latino. Forty-two patients did not specify their ethnicity. Regarding histology, 211 patients were diagnosed with adenocarcinoma and 45 were diagnosed with squamous cell carcinoma. Only three patients had adenosquamous carcinoma while 114 patients were diagnosed with NSCLC-NOS (non-small cell carcinoma – not otherwise specified). The average age at diagnosis was 65.4 years. Thirty-nine and twenty-five patients were diagnosed at Stage I and Stage II, respectively. Sixty-six and one hundred and eighty-six patients were diagnosed with Stage III or IV disease, respectively. Fifty-seven patients had unknown staging. Regarding smoking history, 218 were current or former smokers, while 82 were never smokers. Seventy-three patients’ smoking histories are unknown. Patients who had or were currently smoking had a 42 average pack-year smoking history. A brief summary of patient characteristics is presented in Table 1.

**Table 1:** Patient Characteristics *Due to rounding, percentages may not sum to 100.

N (%)*	The University of Chicago Medicine	NorthShore Health System	Ingalls Health System	Compiled
Total Cases	296 (100)	48 (100)	29 (100)	373 (100)
Sex				
Male	125 (42)	22 (46)	14 (48)	161 (43)
Female	171 (58)	26 (54)	15 (52)	212 (57)
Race				
Caucasian	131 (44)	41 (85)	16 (55)	188 (50)
African-American	109 (37)	3 (6)	12 (41)	124 (33)
Asian	19 (6)	4 (8)	0 (0)	23 (6)
American Indian or Alaska Native	1 (0)	0 (0)	0 (0)	1 (0)
Native Hawaiian or Other Pacific Islander	1 (0)	0 (0)	0 (0)	1 (0)
Unknown	35 (12)	0 (0)	1 (3)	36 (10)
Ethnicity				
Not Hispanic/Latino	250 (85)	48 (100)	29 (100)	327 (88)
Hispanic/Latino	4 (1)	0 (0)	0 (0)	4 (1)
Unknown	42 (14)	0 (0)	0 (0)	42 (11)
Histology				
Adenocarcinoma	155 (52)	44 (92)	12 (41)	211 (57)
Adenosquamous	3 (1)	0 (0)	0 (0)	3 (1)
NSCLC-NOS	100 (34)	0 (0)	14 (48)	114 (31)
Squamous Cell Carcinoma	38 (13)	4 (8)	3 (10)	45 (12)
Average Age at Diagnosis	63.8	66.7	65.6	65.4
Stage at Diagnosis				
I	27 (9)	10 (21)	2 (7)	39 (10)
II	18 (6)	4 (8)	3 (10)	25 (7)
III	55 (18)	5 (10)	6 (21)	66 (18)
IV	154 (52)	15 (31)	17 (59)	186 (50)
Unknown	42 (14)	14 (30)	1 (3)	57 (15)
Smoker				
Yes	179 (60)	12 (25)	27 (93)	218 (58)
No	45 (15)	35 (73)	2 (7)	82 (22)
Unknown	72 (24)	1 (2)	0 (0)	73 (20)
Average Pack Years Smoked	37	40	49	42

### Results of patients who have been tested for EGFR mutations and have taken erlotinib

Of the 373 patients that were gathered, 70 patients had reported data regarding their EGFR mutation status. Out of these 70 patients, 55 (79%) were indicated to have EGFR mutation(s), while 15 (21%) tested negative for mutation(s). The rest of the 303 patients were either not tested for EGFR mutations or their results were unknown. The University of Chicago had 35 patients with at least one EGFR mutation while NorthShore and Ingalls reported 18 and two, respectively. For the wild-type results, the University of Chicago reported seven patients with wild-type EGFR, while NorthShore and Ingalls reported five and three, respectively. Table 2 displays the summary of the number of EGFR tested patients at all institutions.

**Table 2:** EGFR Statuses of Tested Patients *Due to rounding, percentages may not sum to 100.

N (%)*	The University of Chicago Medicine	NorthShore Health System	Ingalls Health System	Compiled
Patients with EGFR Mutation(s)	35 (64)	18 (33)	2 (4)	55 (100)
Patients with Wild-Type EGFR	7 (47)	5 (33)	3 (20)	15 (100)
Unknown/Not Tested	254 (84)	25 (8)	24 (8)	303 (100)

In terms of overall survival, EGFR mutant patients who had taken erlotinib had a median overall survival of 27 months and an average overall survival of 48 months, while their wild-type counterparts had an average overall survival of 36 months and a median overall survival of 26 months. In terms of average duration of erlotinib therapy, patients with EGFR mutations had an average erlotinib therapy of 13 months and a median erlotinib therapy of 12 months, while wild-type patients had an average and median of three months of therapy. A brief summary of overall survival and duration of erlotinib therapy is presented in Table 3.

**Table 3:** Length of Treatment and Overall Survival of EGFR Tested Patients

	Patients with EGFR Mutation(s)	Patients with Wild-Type EGFR
Total Patients	55	15
Average Overall Survival (months)	48 (N=11)	36 (N=11)
Median Overall Survival (months)	27 (N=11)	26 (N=11)
Average Duration of Erlotinib Therapy (months)	13 (N=20)	3 (N=11)
Median Duration of Erlotinib Therapy (months)	12 (N=20)	3 (N=11)

## Discussion

Informatics is an important aspect of current cancer research because it enables researchers to record, analyze, and manipulate an increasingly cumbersome amount of data. Furthermore, it can also facilitate efficient data sharing among research groups. To realize such potential in thoracic oncology research, we formed the Chicago Thoracic Oncology Database Consortium (CTODC), which is composed of research groups from The University of Chicago, NorthShore University Health System, and Ingalls Health System, who have agreed to implement a common database infrastructure and follow common database practices.

To demonstrate the effectiveness of the CTODC’s collective database infrastructure and its data sharing capabilities, we conducted a proof of principle investigation of patients with lung cancer with and without EGFR mutations who had taken erlotinib at the three institutions mentioned previously. The resulting data query resulted in a large number of patients with comprehensive demographical and clinical data from all three institutions of the consortium.

One of the key pieces of data that were analyzed in this investigation was the EGFR statuses of the patients from the consortium. Specifically, the data on the patients’ overall survival and duration of erlotinib therapy showed that patients with EGFR mutations had a longer duration of erlotinib therapy than patients without any EGFR mutations. Similarly, EGFR mutated patients had a longer overall survival compared to the wild-type patients. These results indicate that overall survival and duration of EGFR wild-type and mutated patients on erlotinib are consistent with the published literature.

Overall, the investigation yielded positive results and, in turn, demonstrated advantages of the CTODC. First and foremost, the investigation demonstrated the consortium’s ability to yield large amounts of data from each institution. When data from each institution were centralized for this investigation, it increased the number of cases that would otherwise not be available to individual institutions collecting data from their own databases. This robust data set provides researchers with more ways to detect truer relationships and supported findings. Other benefits the investigation showed were the consortium’s reliability and flexibility to incorporate various database and database platforms. Although the institutions of the consortium followed a set of common core data elements, the institutions have branched off their shared data elements to incorporate different types of data and databases suited to their different interests. One case of this is NorthShore’s history with data collection. At one point in time, the University of Chicago and NorthShore used the Microsoft Access 2010 template as their database. Since both institutions were using the same platform, this made data transmission between the institutions straightforward. However, NorthShore eventually transitioned to the REDCap database. Despite these differing database platforms, the institutions have relatively maintained uniform data elements and smooth data transfer as shown by this investigation.

However, this data sharing model is not without its limitations. First, there is a possibility that patients’ data were duplicated. Specifically, patients’ data from the Northshore University Health System were de-identified before centralization, preventing an otherwise simple task of identifying repeated cases. One way to resolve this issue would be to gather data that would identify cases from the same patient without revealing patients’ personal information. For example, we can update the data collection such that we can verify where each therapeutic agent was administered, solving this particular problem.

Next, there were issues with exporting data. After data were extracted from the consortium’s institutions, there was difficulty combining and comparing shared data elements due to data incompatibility. In one prevalent example, NorthShore encoded their tumor primary site and morphology in a numerical format, while the University of Chicago left these data fields as free text. Therefore, these shared data fields could not be simply combined and analyzed. Either NorthShore or the University of Chicago would have to alter its data in order to fit the format of the other. Although these data compatibility issues were eventually resolved by additional reformatting, one way to address this issue going forward would be to come up with a Microsoft Access VBA script, which would convert the raw data from all institutions into the same format. An additional approach would be to have site visits between institutions in order to monitor changes made to databases and the data. However, the point of implementing databases is for institutions to modify them to suit their research needs. Therefore, there is inevitably going to be discrepancies in the way data is going to be stored and presented at different institutions. While this is inherently not a huge issue since institutions are going to have their own research interests, it does bring up the notion as to how much institutions can deviate their databases from others despite sharing common core data elements. In summary, this issue highlights the need for more constant and efficient communication between institutions whenever a collaborative study or investigation is being performed within the CTODC. The next steps should be taken to improve inter-institutional communication via meetings and guidelines that would 1) allow frequent monitoring by third parties and 2) have institutions commit to making changes in order to improve data collection and transfer. This revised standard operating procedure would further improve the inter-institutional data flow as the CTODC grows to accommodate more institutions and, in turn, more data in the future.

## Conclusions

In this proof of principle investigation, we sought to assess and demonstrate the effectiveness and applicability of the standardization of data collection and sharing within the CTODC. To do this, we examined demographical, clinical, and EGFR molecular data on patients receiving erlotinib at three institutions belonging to the CTODC: the University of Chicago Medical Center, Ingalls Health System, and NorthShore University Health System. For the most part, the investigation was a large success; the institutions were able to effectively share data, resulting in more robust datasets, and demonstrating the reliability and flexibility of the CTODC’s data sharing model. However, this data sharing model has shown some limitations. One key issue we discovered in the investigation was the difficulty of porting and analyzing data. Although this issue was readily resolved with additional effort, it did bring up a concern that a similar situation might occur in the future with additional institutions and their data. To address this issue, we highlighted the need for frequent communication within CTODC so that all institutions of the consortium can commit to changes that need to be implemented, such as employing VBA scripts to fix data porting issues. Consequently, one idea we might venture into would be to improve the CTODC with the suggestions discussed in this investigation and recruit new institutions into the consortium. By increasing the size of the consortium, we can conduct a larger scale investigation to further assess the integrity and effectiveness of the consortium’s ability to collect data.
